# Bad to the Bone: On *In Vitro* and *Ex Vivo* Microbial Biofilm Ability to Directly Destroy Colonized Bone Surfaces without Participation of Host Immunity or Osteoclastogenesis

**DOI:** 10.1371/journal.pone.0169565

**Published:** 2017-01-11

**Authors:** Adam Junka, Patrycja Szymczyk, Grzegorz Ziółkowski, Ewa Karuga-Kuzniewska, Danuta Smutnicka, Iwona Bil-Lula, Marzenna Bartoszewicz, Susan Mahabady, Parish Paymon Sedghizadeh

**Affiliations:** 1 Department of Pharmaceutical Microbiology and Parasitology, Wroclaw Medical University, Wroclaw, Poland; 2 Center for Advanced Manufacturing Technologies (CAMT/FPC), Faculty of Mechanical Engineering, Wroclaw University of Technology, Wroclaw, Poland; 3 Division of Infectious Diseases of Animals and Veterinary Administration, Wroclaw University, Wroclaw, Poland; 4 Department of Clinical Chemistry, Wroclaw Medical University, Wroclaw, Poland; 5 Center for Biofilms and Craniofacial Molecular Biology, Ostrow School of Dentistry of University of Southern California, Los Angeles, California, United States of America; Leibniz-Institut fur Naturstoff-Forschung und Infektionsbiologie eV Hans-Knoll-Institut, GERMANY

## Abstract

Bone infections are a significant public health burden associated with morbidity and mortality in patients. Microbial biofilm pathogens are the causative agents in chronic osteomyelitis. Research on the pathogenesis of osteomyelitis has focused on indirect bone destruction by host immune cells and cytokines secondary to microbial insult. Direct bone resorption by biofilm pathogens has not yet been seriously considered. In this study, common osteomyelitis pathogens (*Staphylococcus aureus*, *Pseudomonas aeruginosa*, *Candida albicans*, and *Streptococcus mutans*) were grown as biofilms in multiple *in vitro* and *ex vivo* experiments to analyze quantitative and qualitative aspects of bone destruction during infection. Pathogens were grown as single or mixed species biofilms on the following substrates: hydroxyapatite, rat jawbone, or polystyrene wells, and in various media. Biofilm growth was evaluated by scanning electron microscopy and pH levels were monitored over time. Histomorphologic and quantitative effects of biofilms on tested substrates were analyzed by microcomputed tomography and quantitative cultures. All tested biofilms demonstrated significant damage to bone. Scanning electron microscopy indicated that all strains formed mature biofilms within 7 days on all substrate surfaces regardless of media. Experimental conditions impacted pH levels, although this had no impact on biofilm growth or bone destruction. Presence of biofilm led to bone dissolution with a decrease of total volume by 20.17±2.93% upon microcomputed tomography analysis, which was statistically significant as compared to controls (p <0.05, ANOVA). Quantitative cultures indicated that media and substrate did not impact biofilm formation (Kruskall-Wallis test, post-hoc Dunne’s test; p <0.05). Overall, these results indicate that biofilms associated with osteomyelitis have the ability to directly resorb bone. These findings should lead to a more complete understanding of the etiopathogenesis of osteomyelitis, where direct bone resorption by biofilm is considered in addition to the well-known osteoclastic and host cell destruction of bone.

## Introduction

Bone infections present unique challenges for treating clinicians, and can be associated with significant morbidity or mortality in affected patients [1]. Bone provides a unique harbor for microorganisms, allowing them to attach to (and colonize) biologic and surgically implanted surfaces while remaining insusceptible to host defenses and antimicrobials [2]. Prolonged courses of antibiotic therapy in conjunction with surgical debridement are mainstays of chronic osteomyelitis therapy in orthopedics [3]. However, antibiotics in general have poor bone penetration and pharmacokinetic bioavailability in bone, which creates additional therapeutic challenges. Furthermore, the causative pathogens of most bone, joint, and prosthetic infections have been shown to grow as biofilm organisms attached to surfaces [4]. These biofilms exhibit greater antibiotic resistance, as well as an altered genotype and phenotype, compared to their planktonic or free-floating counterparts [5]. An analysis of the morbidity and mortality associated with biofilm infections has revealed that each year over 12 million people are affected and 400,000 die as a result of these infections in the U.S. alone [6]. Orthopedic infections are among the most devastating of all biofilm infections due to osteomyelitis and its limb-threatening sequelae, and its prevalence is expected to rise with the increasing partiality for total joint replacement [4].

Virulence factors released by biofilm pathogens during infectious bone disease have been shown to affect host immune cells in addition to bone remodeling cells, culminating in the destruction of hydroxyapatite (HA) [7]. In most cases of chronic osteomyelitis, for example, *Staphylococcus aureus* colonizes bony surfaces and elicits host inflammation and osteoclastogenesis. Research thus far on the pathogenesis of infectious bone disease has focused on this indirect bone destruction by host cells and host factors secondary to microbial insult. The possibility of direct bone or HA resorption by biofilm pathogens during infection has not yet been seriously considered or systematically studied. Direct bone destruction by pathogens *in vivo* may allow invasion of organisms into deeper tissues to evade host immune responses and gain access to eukaryotic cells, or spread hematogenously to reach more viable sites for colonization with access to nutrition [8,9].

We recently hypothesized and demonstrated for the first time that common biofilm pathogens associated with osseous infections can directly destroy HA in the absence of host immunity or osteoclastogenesis *in vitro* [10]. Similar processes have been extensively studied in other hard tissue pathology settings including tooth decay or cariology, in which *Streptococcus mutans* biofilms induce enamel destruction in the absence of host cell activity given the avascular and acellular nature of enamel *in vivo*. Acid liberation from carbohydrate metabolism by this microorganism is the key mechanism initiating enamel dissolution [11]. Recent insight into bone diagenesis gained from the fields of paleoecology and paleopathology further demonstrate the importance of direct microbial degradation of hard tissues, i.e. bone and teeth [12]. Bone diagenesis has been shown to be a complex and site-specific process; and it is dependent on various external features of the burial environment such as pH, temperature, presence of oxygen, soil composition, groundwater chemistry, and most prominently, microbial-induced bioerosion [12]. Finally, accumulating evidence from the fields of environmental microbiology and marine microbiology also demonstrate the important role of biofilms in damaging colonized biotic and abiotic surfaces, thus contributing to the deleterious effects such as biofouling (microfouling) and biocorrosion [13].

Our central hypothesis is that the difficult management of infectious bone disease could be materially improved by the adoption of concepts and methods that have been useful in the study and treatment of biofilms in environmental, paleopathological, and other seemingly unrelated microbiological systems of medicine and dentistry. Therefore, in this line of investigation, we expand on previous work aimed at understanding the nature and direct role of microbial biofilms in infectious bone disease pathophysiology and HA destruction. To this end, we designed and conducted several *in vitro* and *ex vivo* experiments to study various quantitative and qualitative aspects of bone destruction during biofilm-mediated infection.

## Materials and Methods

### Experimental strains

The following ATCC collection strains were chosen for experimental purposes: *S*. *aureus* 6538, *P*. *aeruginosa* 15442, *S*. *mutans* 25175, and *C*. *albicans* 10231.

### HA discs

Commercially available HA powder was used for custom disc manufacturing. Powder pellets of 9.6mm diameter were pressed without a binder. Sintering was performed at 900°C. The resulting tablets were compressed using the Universal Testing System for static tensile, compression, and bending tests (Instron model 3384; Instron, Norwood, MA). The quality of the manufactured HA discs was checked by confocal microscopy and micro–computed tomography (microCT) using a LEXT OLS4000 microscope (Olympus, Center Valley, PA) and Metrotom 1500 microtomograph (Carl Zeiss, Oberkochen, Germany).

### Rat jaws

Wistar strain male rats weighing 300-350g were used in this study. Rat mandibles were resected postmortem. The animal material was obtained from a research project titled “Cardioprotective effect of inhibitors of matrix metalloproteinase-2 and inhibitors of post-translational modifications of myosin light chain 1, 2 on function of heart during ischemia/ reperfusion-induced injury”. In the aforementioned study, rats were sacrificed by intraperitoneal application of pentobarbital (40mg/kg), followed by thoracotomy and heart excision. Drugs or other procedures, which could potentially influence bone structure, were not used or performed during rat inbreeding. To obtain HA surface for biofilm development, mandibular soft tissues were surgically removed. Subsequently, these jaws were antiseptically rinsed using saline and UV-irradiation. Jaws prepared in this manner were frozen at -80°C for further analysis. To test jawbone sterility, three jaws were incubated in anaerobic conditions in thioglycollate broth for 7 days in accordance with microbiological procedures for anaerobic organism culturing. Another three jaws were incubated for 2 days in aerobic conditions in brain-heart infusion (BHI) media.

### Ethics statement

This investigation conforms to the Guide to the Care and Use of Experimental Animals published by the Polish Ministry of Science and Higher Education. Our study was approved by the local Ethics Committee for Experiments on Animals at the Ludwik Hirszfeld Institute of Immunology and Experimental Therapy, Polish Academy of Sciences, Wroclaw, Poland (Protocol #34/2014).

### Media

Biofilms were grown in the following microbiological media: Tryptic-Soya Broth (TSB) for *S*. *aureus*, *P*. *aeruginosa*, *C*. *albicans*, and BHI media for *S*. *mutans* and mixed biofilm of *S*. *mutans + C*. *albicans*. The strains were also incubated in the aforementioned media implemented with 3% sucrose, and in artificial saliva (AS) with or without the addition of 3% sucrose. The sugar supplement was added to media to simulate conditions where bone in the oral cavity is exposed and immersed with liquid, such as saliva. Artificial Saliva (AS) was composed of: 2.5 g l−1 mucin, 0.25 g l−1 sodium chloride, 0.2 g l−1 potassium chloride, 0.2 g l−1 calcium chloride, 2.0 g l−1 yeast extract, 5.0 g l−1 protease peptone, and 1.25 ml l−1 40% urea; AS was adjusted to pH level 6.

### Confirmation of absence of eukaryotic live cells on analyzed bone material

To exclude any eukaryotic cellular activity in rat jaws, fluorescence microscopic imaging and live-dead assessments were applied. The PDV mouse keratinocyte cell line served as the positive control for this experiment (living cells), while PDV mouse keratinocyte cell line killed with the 3.7% formaldehyde served as the negative control (dead, fixed cells). Rat jaws served as the experimental sample. PDV mouse keratinocyte cell line was cultured on glass cover slips in DMEM medium supplemented with 10% fetal bovine serum and antibiotics for 48 hrs until 80% confluency. The cells were fixed with 3.7% formaldehyde for 10 min at room temperature or left untreated. Next, live and fixed (dead) cells were incubated with SYTO-9 (3.34 uM) and Propidium Iodide (PI, 20 uM) diluted in PBS buffer for 10 min at room temperature. Imaging was performed in HBSS medium at room temperature on a Leica SP8 confocal microscope with a 25x water dipping objective using 488 nm laser line and 500–530 nm emission to visualize SYTO-9 and 552 nm laser line and 575–627 nm emission to visualize PI, in a sequential mode. Images are maximum intensity projections obtained from confocal Z stacks with 2 um spacing in Z dimension. PI is represented in red, SYTO-9 in green. Rat jaws were processed and imaged in the same fashion.

### Biofilm formation on rat jaws, HA discs, and polystyrene

Strains cultured on appropriate agar plates (*S*. *aureus*, Columbia plate; *C*. *albicans*, Sabouraud plate; *S*. *mutans*, BHI plate; *P*. *aeruginosa*, MacConkey plate) were transferred to liquid microbiological media as described in the previous section and incubated for 24 hours at 37°C under aerobic conditions, with the exception of *S*. *mutans*, which was incubated under 5% CO_2_ supplemented atmosphere. After incubation, strains were diluted to the density of 1 McFarland (MF). The microbial dilutions were inoculated to wells of 12-well plates containing rat jawbones or HA discs as a substrate, or simply to polystyrene wells in which case the bottom surface of the wells served as the substrate for biofilm development. Strains were incubated for 4 hours at 37°C, followed by removal of the microbe-containing solutions from the wells. The surfaces of the jaws, HA discs, and polystyrene plates were gently rinsed to leave adhered cells and remove planktonic or loosely-bound microbes. Surfaces prepared in this manner were immersed in fresh, sterile media and left for 7 days. Every second day, half of the media was removed and replaced with fresh aliquot to provide biofilm nutrients and decrease potential impact of planktonic cells on the surfaces analyzed. To obtain a mixed biofilm, 1 MF of *S*. *mutans* and 1 MF of *C*. *albicans* was combined and incubated for 7 days on the three aforementioned substrates similar to the single-species biofilm.

### Confirmation of biofilm formation by quantitative cultures

Microbial dilutions were incubated on surfaces for 7 days at 37°C as previously described. After incubation, the surfaces were rinsed using physiological saline solution and transferred to 1 mL of 0.5% saponin (Sigma-Aldrich, St. Louis, MO). The surfaces were vortex-mixed vigorously for 1 minute to detach cells. *S*. *mutans* biofilm and mixed *S*. *Mutans* + *C*. *albicans* biofilm grown in media supplemented with 3% sucrose formed plaque structures partially resistant to the aforementioned type of treatment. Therefore, plaque was gently removed from surfaces with a woolen swab, introduced to 1 ml of 0.5% saponin and shaken for 1 minute to mechanically detach the *S*. *mutans* extracellular polysaccharide biofilm matrix. Next, the solution was diluted 10 times with 0.9% NaCl to neutralize saponin activity. Surfaces with partially removed *S*. *mutans* biofilm were simultaneously subjected to the same procedure as other strains. The two solutions were combined and centrifuged, and the cell-containing pellet was dissolved in 1 ml BHI media. Subsequently, all microbial suspensions were diluted 10−10^9^ times. Each dilution (100 mL) was cultured on the appropriate stable medium (*S*. *aureus*, Columbia; *C*. *albicans*, Sabouraud; *S*. *mutans*, BHI; *P*. *aeruginosa*, MacConkey) and incubated at 37°C for 24 hours. After 24 hours, the microbial colonies were counted and the number of cells forming biofilm was assessed. All measurements were repeated in triplicate. Results were presented as the mean number of colony-forming units per square millimeter surface ± standard error of the mean. To estimate the exact surface area of HA discs and jawbones, x-ray tomographic analysis was applied in a manner described in the Materials and Methods subheading “Quantitative assessment of alterations in bone structure caused by S. aureus evaluated by micro-computed tomography”. For estimation of the area of test plate bottoms, the equation for circle area (πr^2^) was applied.

### *S*. *aureus* biofilm formation during 30-day incubation and microbiological purity analysis

This experiment was performed in conditions as described in the previous section, but biofilms were grown for an extended period of 30 days instead of a 7-day period. Microbiological media was changed 15 times during this portion of the experiment; consequently, microbiological analysis was performed every other day (as in the 7-day experiment) in order to find potential contamination with microorganisms other than *S*. *aureus*. Therefore, 500 μL of bacteria-containing solution was taken from test wells and examined using standard Gram staining and light microscope imaging under magnification X1000 (100X objective x 10 immersion oil) to evaluate for cells other than cocci. Furthermore, another 500 μL of solution was introduced to 3 mL of BHI media and left for 24 hours at 37°C in aerobic conditions, and another 500 μL in thioglycollate broth for 7 days according to microbiological procedures of anaerobic organism culturing. After the incubation period, bacteria-containing solutions were examined again using the Gram staining technique and cultured on appropriate stable selective media to exclude presence of microbes other than *S*.*aureus*.

### Confirmation of biofilm formation by scanning electron microscopy

After incubation as described in Materials and Methods subheading “Confirmation of biofilm formation by quantitative culture,” surfaces were rinsed using physiological saline solution to remove non-adherent organisms and leave only biofilm-forming structures. Subsequently, the discs were fixed using 3% glutarate (Poch, Gliwice, Poland) for 15 minutes at room temperature. Samples were rinsed twice with phosphate buffer (Sigma-Aldrich Poland, Poznan, Poland) to remove the fixative. Dehydration in increasing concentrations of ethanol (25%, 50%, 60%, 70%, 80%, 90%, and 100%) was performed for 10 minutes per solution. The ethanol was then rinsed off, and samples were dried at room temperature. Next, the samples were covered with gold and palladium (60:40; sputter current, 40 mA; sputter time, 50 seconds) using a Quorum machine (Quorum International, Fort Worth, TX) and examined under a Zeiss EVO MA25 scanning electron microscope (SEM) (Carl Zeiss, Oberkochen, Germany). Strains were considered competent to form biofilm if they were able to adhere to the HA surface and if they were at least partially embedded within the extracellular biofilm matrix. Moreover, Energy Dispersive Spectroscopy (EDX) technique was performed to confirm that portions of material observed in SEM images consist of hydroxyapatite and to support the hypothesis that these materials are due to bone loss caused directly by microbes. For this purpose, positive control samples (consisting of pure HA) and negative control samples (soft tissue scrapped from rat jaws) were used and their element composition was compared to element composition of bone alterations analyzed.

### pH assessment

Each day of the experiment period, 100 μL of bacteria-free solution from plate wells containing analyzed surfaces was taken from the 24-well plates and analyzed using a universal pH indicator (Merck, Warsaw, Poland).

### Biofilm removal from analyzed surfaces

Microbial biofilms (grown under conditions specified previously in Materials and Methods subheadings “Biofilm formation on rat jaws, HA discs, and polystyrene” and “*S*. *aureus* biofilm formation during 30-day incubation and microbiological purity analysis”) were removed from surfaces analyzed in the manner described in our previous work [10]. Briefly, HA discs or rat jawbone surfaces were transferred to new wells of test plates and rinsed thoroughly with ultrapure water. Next, rinsed discs were transferred to new plates filled with 2 mL of 10% saponin detergent (Sigma-Aldrich, Poland) and left for 20 minutes at room temperature. After incubation, plates were mounted onto a plate shaker (Schuttler MTS 2; Ika, Wilmington, SC) for 2 minutes at a speed of 1,000 per minute. The activity of saponin detergent combined with intensive shaking led to complete removal of biofilm from analyzed surfaces. This procedure was performed for all strains examined, with the exception of *S*. *mutans* and *S*. *Mutans + C*. *albicans* mixed biofilm grown in media supplemented with 3% sucrose. These biofilms formed plaque structures partially resistant to the aforementioned type of treatment. Therefore, the plaque was subjected to 20 minutes of incubation with 10% saponin, and then removed gently with a woolen swab. Subsequently, discs with partially removed plaque were subjected to shaking for 2 minutes at a speed of 1,000 per minute. After shaking, discs were rinsed thoroughly with ultrapure water and dried at room temperature. For control purposes, 3 HA discs and 3 rat jaws were then introduced to appropriate liquid media and left for 24 hours at 37°C. After this time, 1% 2,3,5-triphenyl-2H-tetrazolium chloride (TTC) was added to the medium. TTC is a colorless compound that changes into red formazane in the presence of living, metabolically-active microbial cells. Lack of color change confirms complete biofilm removal. Moreover, the sterile HA discs and rat jaws were incubated for 7 or 30 days with media implemented with antibiotic mixture (penicillin and streptogramin) and antifungal (amphotericin B) to avoid potential contamination. These sterile surfaces were treated with saponin and vortexed in the same manner as surfaces covered with biofilm, and served as control samples to assess possible effects of incubation in media and saponin/vortex treatment on analyzed surfaces.

### Quantitative assessment of alterations in bone structure caused by *S*. *aureus* evaluated by micro-computed tomography

For the purposes of this experiment, *S*. *aureus* biofilms were selected. The structure and surfaces of jawbones were assessed using micro-CT methodology (Metrotom 1500 microtomograph, Carl Zeiss, Oberkochen, Germany). The analysis was performed using VG Studio MAX software (Volume Graphics, Heidelberg, Germany). After processing of data segmentation using a local thresholding method, volume models representing reconstructed osseous geometry were applied to obtain analyzed bone surfaces and volume. The bones were UV-irradiated and used as a surface for *S*. *aureus* biofilm formation for 7 or 30 days as described previously. After incubation, jawbones were subjected to the aforementioned method of biofilm removal and subjected to another round of micro-CT analysis to compare bone geometry after biofilm development. The bones were incubated with sterile media containing an antibiotic mixture (penicillin and streptogramin) and an antifungal (Amphotericin B); then processed for biofilm removal to serve as controls for analysis. To quantitatively compare changes of geometry, samples were adjusted using the least-squares method, which allows analysis of geometric deviation. Sample volumes were also calculated with this method. Bone volumes for biofilm versus control samples were statistically compared using ANOVA with significance accepted at p<0.05.

### Statistical analysis

Statistical calculations were performed with the SigmaStat package, Version 2.0 (SPSS, Chicago, IL). A power analysis was performed to determine sample size estimation prior to experimentation. Quantitative data from experimental results was analyzed using the Kruskall-Wallis test, post-hoc Dunne’s test, or ANOVA, as applicable, and statistical significance was accepted at p <0.05.

## Results

### Confirmation of absence of eukaryotic live cells on the analyzed bone material

**Fig 1:**
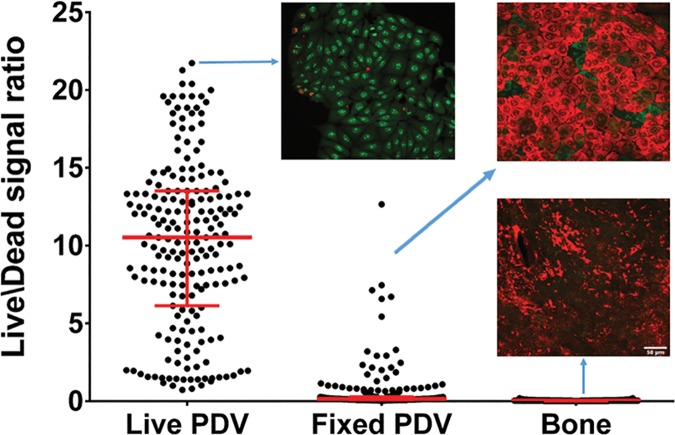
Confirmation of absence of eukaryotic live cells in analyzed bone material. Graph shows live/dead ratio values determined for cells and bones, horizontal bar is median and whiskers represent interquartile range. Differences in live/dead ratio are statistically significant, all medians vary between themselves significantly (Kruskal-Wallis test, p<0.05). Live PDV–positive control sample; green color confirms activity of live eukaryotic cells; Fixed PDV–negative control sample; red color confirms lack of live eukaryotic cells. Bone–analyzed material; red color confirms lack of live eukaryotic cells in analyzed rat jaws.

This was performed using fluorescence microscopic imaging with live/dead assessment. As shown in **Fig 1**, there are no signs of eukaryotic cell activity on the investigated rat jaws. Therefore, all bone alterations observed and analyzed further in this manuscript were a result of direct microbial activity.

### SEM biofilm analysis

**Fig 2:**
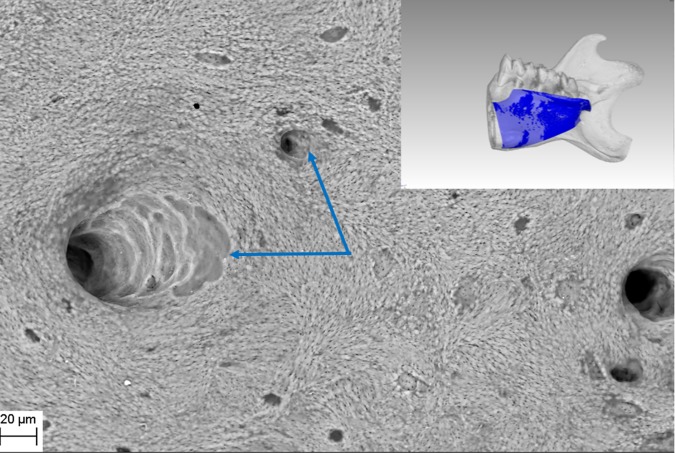
Native structure of rat jawbone showing characteristic surface morphology and lacunar-canalicular systems (arrows), as viewed under scanning electron microscopy (Magn.811X, SEM Zeiss EVO MA25 microscope). Right inset shows gross resected rat mandible used in this study and the lingual-bone region (blue) used for biofilm inoculation.

**Fig 3:**
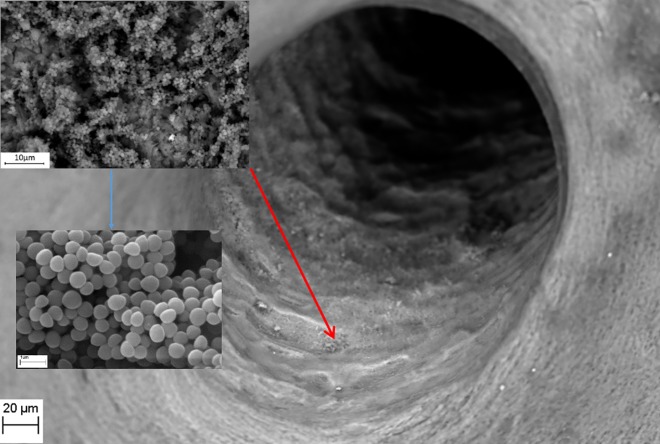
*S*. *aureus* ATCC6538 biofilm (upper left inset, magnification 4750X) formed inside a native bone canal (red arrow; Magn.811X). Higher magnification (blue arrow, lower left inset Magn.14560X) reveals multi-layer composition of staphylococcal biofilm. Media: artificial saliva plus 3% sucrose. SEM Zeiss EVO MA25 microscope.

**Fig 4:**
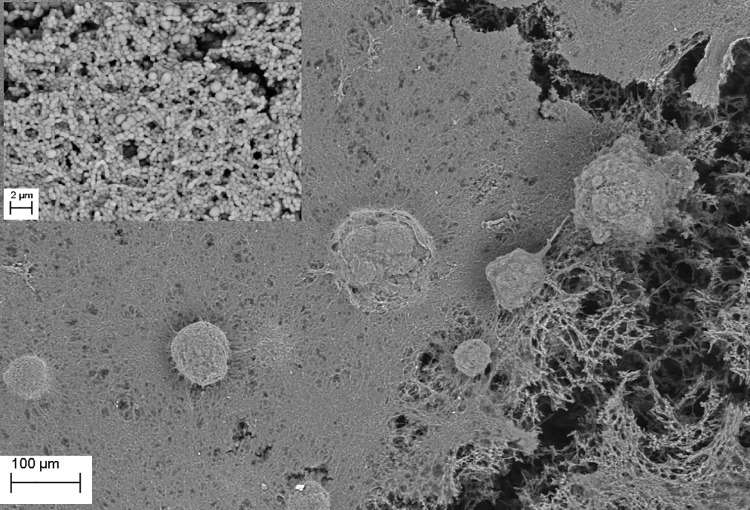
*S*. *mutans* ATCC25175 biofilm (plaque) formed on the surface of bone (Magn.275X). The dense structure of plaque is seen in the right and central part of the image, whereas plaque disruption is visible in the right portion of the image revealing multilayer composition and biofilm matrix (upper left inset, Magn. 660X). Media: artificial saliva plus 3% sucrose. SEM Zeiss EVO MA25 microscope.

**Fig 5:**
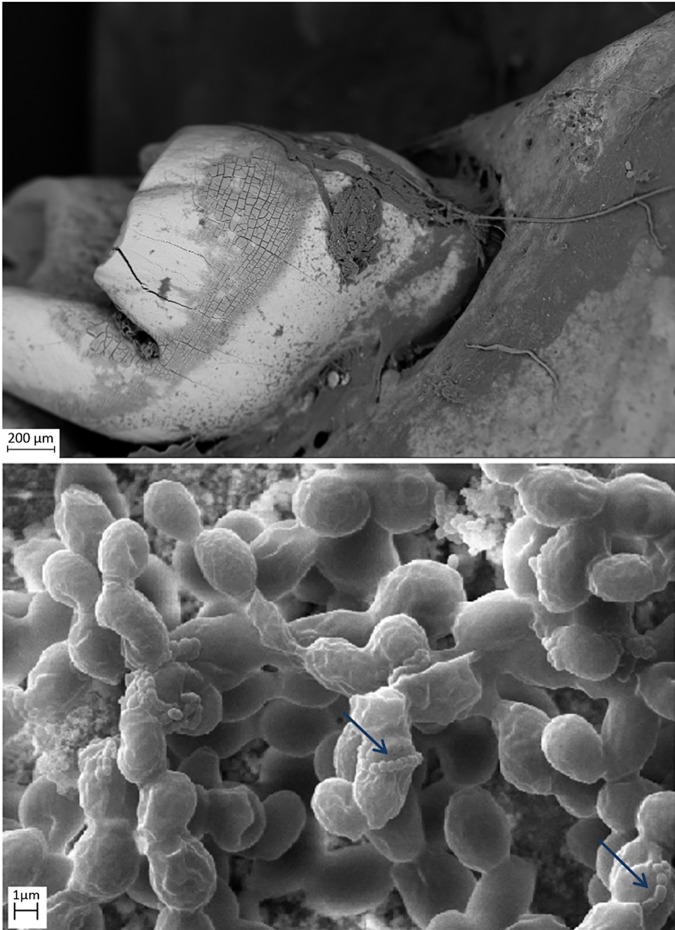
*S*. *mutans* ATCC25175 and *C*. *albicans* 10231 mixed species biofilm formed on jawbone surface subjacent to a tooth (upper image Magn.95X; lower image Magn.9850X). Note: streptococci co-aggregation with candida cells (blue arrow). Media: artificial saliva. SEM Zeiss EVO MA25 microscope.

**Fig 6:**
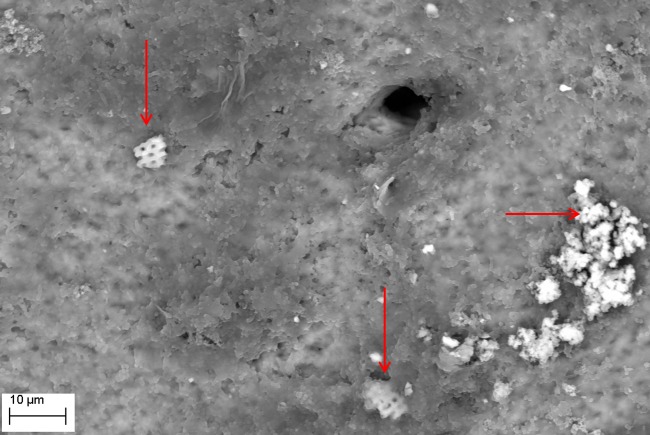
*P*. *aeruginosa* ATCC15442 biofilm formed on jawbone surface (Magn.2570X). Note: fragments of detached bone indicated by red arrows. Elemental composition of these alterations was experimentally confirmed. Media: artificial saliva plus 3% sucrose. SEM Zeiss EVO MA25 microscope.

**Fig 7:**
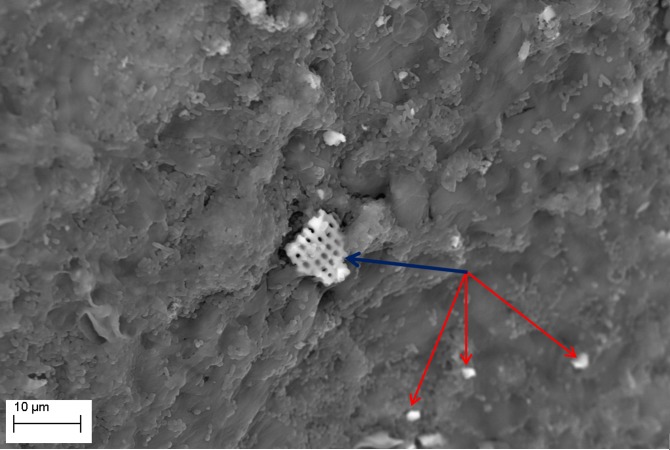
*S*. *mutans* ATCC25175 biofilm formed on jawbone surface. Note: larger fragment of detached bone indicated by the blue arrow, and smaller fragments indicated by red arrows. Media: artificial saliva. Magn.2990X, SEM Zeiss EVO MA25 microscope.

**Fig 8:**
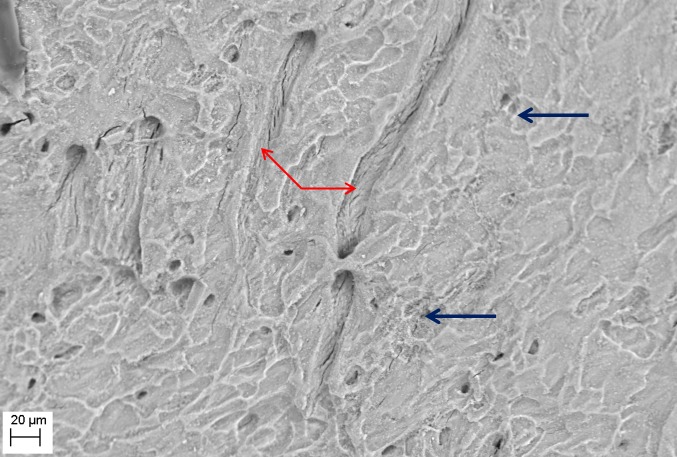
Removal of *P*. *aeruginosa* ATCC15442 biofilm from bone reveals characteristic surface alterations trails (red arrows) and cavities (blue arrows); as compared to Fig 1 representing intact bone structure, and Fig 9 representing the process of cavity and trail formation. Media: artificial saliva plus 3% sucrose. Magn.645X, SEM Zeiss EVO MA25 microscope.

**Fig 9:**
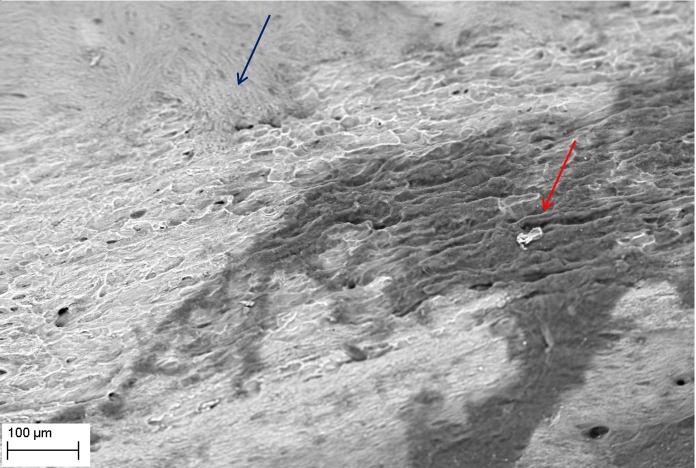
Removal of *C*. *albicans* 10231 biofilm from bone surface. Note: two types of bone structure are indicated; intact (blue arrow), and altered (red arrow). Media: artificial saliva plus 3% sucrose. SEM Zeiss EVO MA25 microscope.

SEM evaluation showed that all tested biofilm strains, single species as well as mixed species, were able to form mature biofilms within 7 days on all analyzed surfaces (HA discs, rat jawbone, and polystyrene wells) regardless of the media applied. **Fig 2** shows native control jawbone with characteristic lacunar-canalicular systems, and representative biofilm colonization of the bone is shown in **Figs 3–7**. All tested strains (*Staphylococcus aureus*, *Pseudomonas aeruginosa*, *Candida albicans*, *Streptococcus mutans*, and mixed biofilm of *S*. *Mutans + C*. *albicans*) formed mature multicellular biofilm structures. Most of the cellular structures formed on the various tested substrates were embedded within the extracellular matrix, representing the different stages of biofilm formation from early to mature matrix-forming biofilms. In the mixed species biofilm, microbiologic co-aggregation or cell-to-cell recognition of genetically distinct cell types, which is thought to contribute to biofilm pathogenicity [14], was observed between experimental yeast and bacteria (**Fig 5**). Removal of biofilms after each experimental period revealed alterations of bone surfaces, as indicated in **Figs 8 and 9,** for all samples as compared to controls. This finding was similar to that observed in our previous work [10] where HA discs were used for biofilm growth.

**Fig 10:**
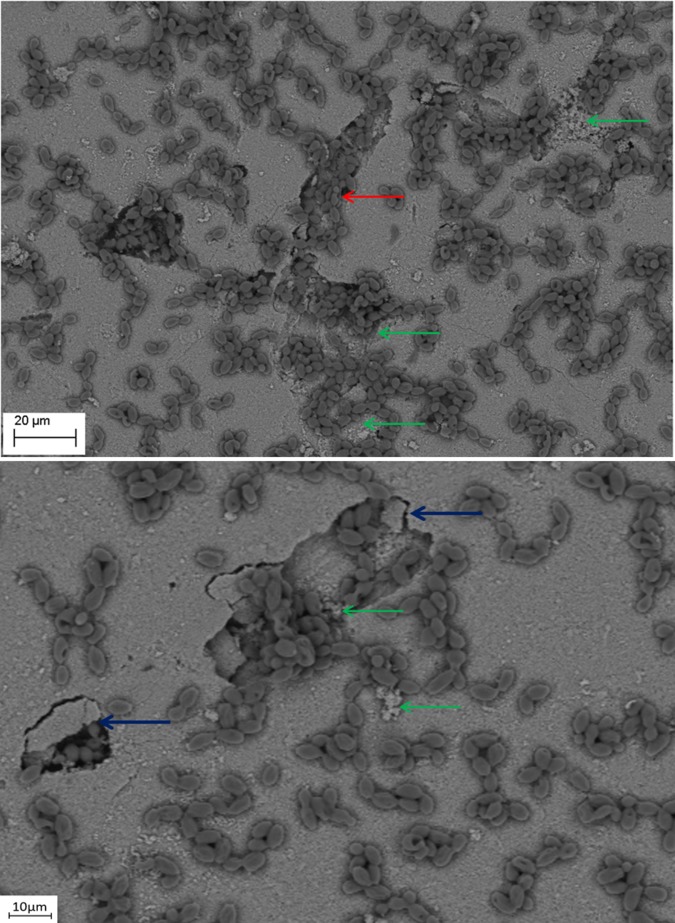
*C*. *albicans* 10231 cells remodeling hydroxyapatite surface. Magn. 1300X (lower image) and 1010X (upper image). The process of cavity and sequestrum formation is indicated by blue arrows. Formation of trails resembling those seen in **Figs 7 and 8** is marked with a red arrow. Destruction of hydroxyapatite is shown with the green arrow. Please see **Fig** 13 for potential mechanism of sequestrum formation. Media: artificial saliva plus 3% sucrose. SEM Zeiss EVO MA25 microscope.

The presence of detached fragments of bone similar to sequestra was observed frequently on top of mature biofilm structures as shown in **Figs 6 and 7**. EDX analysis revealed that these fragments consist only of the following elements: Ca– 22.74%; P– 10.64%; O– 66.62% and this pattern correlated strongly with the pattern obtained experimentally for pure hydroxyapatite (Ca– 35.84%; P– 18.25%; O– 45.91%), which is consistent with spectroscopic patterns for HA/bone. During observed biofilm growth, portions of HA became loosely adherent and formed partially-covered cavitations. During biofilm maturation, microorganisms invaded the cavity and displaced loosely adherent HA fragments forming sequestrum-like structures. Subsequently, these cavities extended over time to become long and narrow trails formed by growing biofilms. Characteristic histomorphologic changes of sequestrum formation and bone destruction by biofilm pathogens is indicated in **Fig 10**. Detached bone fragments seen in this Figure were similar to sequestrum seen clinically and histopathologically in osteomyelitis specimens; thus, we postulated that this phenomenon may provide insight into potential mechanisms of bone destruction. Therefore, we performed experiments using HA discs and polystyrene controls similar to those performed on actual jawbone. The rationale being that biologic bone has intrinsic irregularities and heterogeneity on a microscopic level, which can potentially lead to false positive results or inaccurate quantitative morphometric calculations concerning microbial ability to destroy and alter bone surface. Our customized HA discs are fabricated, calibrated, and measured to have high surface smoothness and homogeneity, as shown in our previous study [10].

### pH assessment

The biofilms of tested microbes were grown on HA discs, jawbones, or polystyrene in rich media (TSB, BHI, or their variants implemented with 3% sucrose) or poor media (saliva or saliva implemented with 3% sucrose). The aim of this experimental setting was to establish whether different media and substrates have an impact on acid-base status, and to understand the role of pH as a mechanism for bone dissolution. Results from our experiments indicated that both media and substrate have a significant impact on pH levels produced by biofilms, as shown in **S1 File**. In the case of *S*. *aureus* biofilms formed on bone, supplementation of saliva with sucrose led to an increase in the pH level by 1 unit, while in the case of TSB media, additional sugar content increased the pH level by 2 units. On the contrary, in the case of *S*. *aureus* biofilms formed on polystyrene, the addition of sucrose decreased pH levels. The pH of *S*. *mutans* was always acidic, independent of surface or media applied; however, the lowest pH values were observed for BHI media with the addition of sucrose. The pH measured for *P*. *aeruginosa* biofilm was generally above level 7, and it reached its peak (level 10) with saliva plus 3% sucrose and biofilm grown on HA discs. The pH values measured for *C*. *albicans* were also above 7, with the exception of biofilm grown in TSB media plus 3% sucrose, where the pH values were 5–6 for HA discs and polystyrene. Interestingly, in the case of *C*. *albicans* biofilms formed on jawbones, pH values for TSB plus 3% sucrose were basic. These findings confirm the role and high complexity of interactions between specific species of microorganisms, various media, and substrates on acid-base status.

### Quantitative cultures

**Fig 11:**
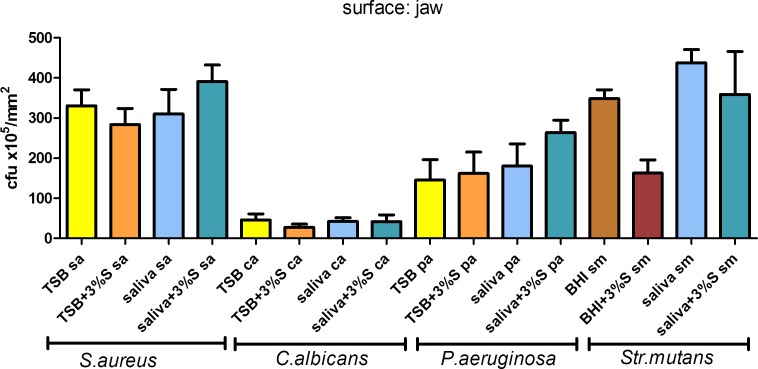
Number of cells forming biofilm on jaws in TSB, TSB media supplemented with 3% sucrose; artificial saliva, saliva supplemented with 3% sucrose; BHI; BHI supplemented with 3% sucrose.

To assess the number of cells forming biofilm on jawbone and controls, quantitative cultures were performed. Although time-consuming, this technique is considered one of the most useful for this purpose. The main aim of this part of the experiment was to assess the correlation between media applied and the potential of examined microbes to form and develop biofilm structure on these specific structures. The application of micro-CT allowed us to standardize results obtained from different surfaces, and to present them in the form of colony forming units per mm^2^ of surface. The average surface area of tested jawbones, HA discs, and plate bottoms was 308±64mm^2^, 198±0.35mm^2^, and 200mm^2^, respectively. All species were able to adhere to tested surfaces. The results of quantitative cultures from rat jaws, grouped with respect to medium applied and pathogen, are presented in **Fig 11**, while results of quantitative culture from HA discs and polystyrene are grouped and shown in **S2 File**. Results indicate that media and substrate had no meaningful impact on biofilm formation, and observed differences in cell number were statistically insignificant (Kruskall-Wallis test, post-hoc Dunne’s test; p <0.05).

### Quantitative assessment of *S*. *aureus*-induced alterations in bone structure by micro-computed tomography

**Fig 12:**
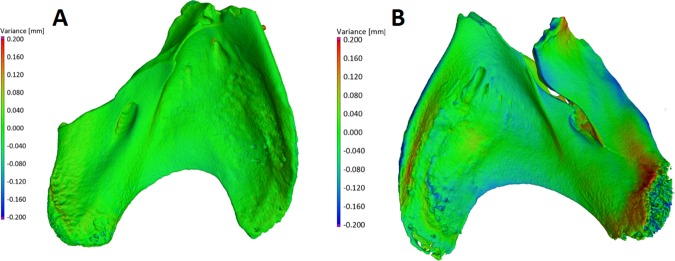
The geometry of control bone sample (**A,** left side of Figure), incubated for 30 days in sterile media and geometry of treated bone sample (**B**, right side of Figure), which was used as a surface for *S*. *aureus* biofilm growth. The green surface represents position points which did not change significantly from day 0 to day 30. The colors from light blue to violet represent points which collapsed during 30 days in comparison to day 0, and colors from orange to red represent points which were higher after 30 days of incubation than the same points in the beginning of experiment (day 0).

The aim of this experimental setting was to check parametrically the ability of microorganisms to alter bone surface morphology. In previous experiments presented in this line of investigation, we indicated that experimental species were able to adhere to bone, form biofilm, and survive in various nutritional conditions [10]. We now demonstrate that biofilm growth on different microbiological media and surfaces correlates with strong pH fluctuations. We also observed the phenomenon of cavity formation by biofilms over time, supporting the concept of microbial ability to decay bone without participation of immune effector cells or osteoclasts. To quantitatively assess impact of biofilm on bone structure, we chose the pathogen *S*. *aureus* as it is the most common bone pathogen in osteomyelitis, and its biofilm is easily removed during *in vitro* experiments (contrary to *S*. *mutans* plaque removal, which requires additional procedures that can result in false positive results). Moreover, as we demonstrated with pH assessments in TSB media, values of pH for *S*. *aureus* biofilm vary from levels 6 to 8 providing both acidic and basic conditions. Initially, we performed micro-CT assessments for bone samples incubated for 7 days; however, results obtained were on the border of apparatus sensitivity. Therefore, we prolonged the time of jaw incubation with *S*. *aureus* to 30 days in order to establish chronic and mature biofilms as this is more relevant to clinical conditions. Micro-CT results of biofilm impact on jawbone are presented in **Fig 12**. The measurement error for this method was 3.13μm. Our method covered 90% of sample surface area. For control bone samples, average change in geometry after 30 days of incubation in sterile media was 28.5μm±3.9 μm. The average difference in geometry of bone samples incubated with *S*. *aureus* biofilm was over 3 fold higher and equaled 98.16±3.6 μm. The application of micro-CT allowed us to also assess changes in bone volume. At least one mechanism for bone detachment (cavity and sequestrum formation) was observed in this work, thus we hypothesized that the presence of biofilm on bone samples might lead to bone volume reduction. Average bone volume in the beginning of experimentation was 43.89mm^3^±8.4 mm^3^. The 30 day incubation of bone in sterile media had little impact on its volume (decrease by 1.22±0.6% of total volume). The presence of *S*. *aureus* biofilm on bone samples led to a decrease of total volume by 20.17±2.93%, which was statistically significant as compared to controls (p <0.05, ANOVA).

## Discussion

In this study, we highlighted a possible direct role of microbial bone damage. We found dynamic and characteristic changes to bone and bony destruction caused by biofilms that were similar to those observed *in vivo* or in clinical specimens of osteomyelitis histopathologically [4,15]. All tested biofilm pathogens were able to destroy and alter bone in the form of cavities and trails, and in the absence of host immune cells or osteoclasts. Bone volume was significantly reduced by infecting biofilms as compared to controls, supporting bone destruction by pathogens. All tested biofilms demonstrated significant quantitative and qualitative damage to bone. The pathogenesis of bone destruction in osteomyelitis to date has been considered mainly a result of secondary host activity and osteoclast-mediated cavitation and trail formation [6,16], with no direct contribution by pathogens in the bone dissolution process. However, our findings indicate that biofilm pathogens associated with infectious bone disease have the ability to directly resorb bone and cause characteristic alterations similar to those seen clinically and histopathologically in osteomyelitis.

**Fig 13:**
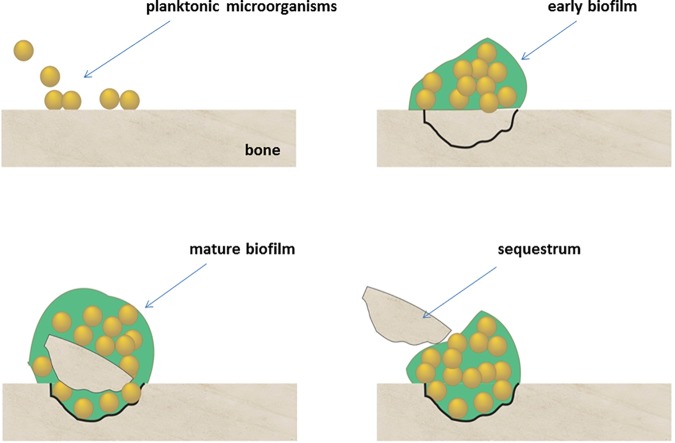
This schematic illustrates the development of sequestrum over time as planktonic organisms attach to bone and form mature biofilms, leading to bone cavitation and eventual detachment of infected fragments.

In patients with chronic osteomyelitis, for example, segments of nonvital bone become separated and form sequestra that can continue to harbor biofilm microbes despite antibiotic treatment; and because antibiotics and inflammatory cells cannot reach avascular sequestra clinically, conservative medical treatment fails [4]. Sequestrum formation is a characteristic feature of chronic osteomyelitis, and was observed to develop over time in our model system with biofilm maturation, as illustrated in **Fig 13**. Our findings that microbes tested grew robustly in all variants of experimental settings confirm the theory that common bone pathogens are resilient biofilm organisms that are able to adapt and thrive on various surfaces and nutritional conditions, and can cause damage to those colonized structures. Similar findings have been observed in bone diagenesis research where investigators found that postmortem bones from complete burials showed greater erosion, due to bacterial attack, than fragmented bones resulting from dismemberment or butchering [17]. In marine and environmental biofilms, the enhanced and sometimes unique ecophysiological activities of surface or biofilm-associated microbial communities lay the foundations for biogeochemical functions that can sharply differ from those of free-living (planktonic) microbial communities [18]. For example, biofilm-associated microbial communities may thrive in extreme or hostile environments where individual microorganisms would find the maintenance of activity, growth, or even survival, to be challenging [13].

To understand potential mechanisms of bone dissolution during biofilm-mediated infection, we also studied pH changes in experimental biofilms over time, and found that all strains were able to change the acid-base status of their environment depending on the type of substrate or media applied. In other biofilm-mediated infections, investigators have shown that small changes in pH from bacterial metabolism may influence the activity of proteolytic enzymes of host and bacterial origin [19]. In cariology research, the adverse effect of acidic pH on tooth dissolution caused by oral bacteria has been clearly demonstrated [19]. The correlation between low or acidic pH levels and adverse effects on bone health has also been previously established; however, data from bone diagenesis research indicates that higher or basic pH values can have damaging effects on bone health [12]. We also observed such a phenomenon in our previous study, and found that both acidic and basic environments produced by bacteria were still associated with HA damage [10]. Organisms from the lowest or deepest layers of a biofilm community grow in conditions of low oxygen tension, which can cause activation of anaerobic metabolic processes of carbohydrate digestion and release of acidic byproducts such as lactic and ferric acid. The impact of the substrate serving as a basis for biofilm development also affects biofilm cell metabolic activity and pH, in addition to biofilm abundance, architecture, and extracellular matrix composition [20]. Results from our experiments support previous data that substrate and media have a significant impact on pH levels produced by biofilms and biofilm growth over time.

The extent and clinical significance of direct bone resorption by biofilms remains unclear and requires further investigation. In this study we observed direct bone destruction due to biofilms and we proposed mechanistic possibilities for this phenomenon. However, a comprehensive analysis of virulence factors that might be responsible for observed sequestrum formation still needs to be performed and was outside the scope of the current study. Cassat et al highlighted *S*.*aureus* virulence factors that were involved in osteomyelitis in a murine model [21]. Exoproteome analysis performed in the above-mentioned study revealed a key role of Sae-regulated protease referred to as aureolysin in addition to other osteolytic peptides in triggering osteoblast cell death and bone destruction. However, this area still needs to be further investigated due to environment-related variability in expression of *S*.*aureus* virulence factors. On the contrary, *Str*.*mutans* virulence factors that are responsible for dental carries and bone damage are relatively well-recognized. The most well-known factors responsible for dental carries are *Str*.*mutans* exopolysaccharides (EPS), and EPS production might be sucrose-dependent or sucrose-independent. Other meaningful virulence factors of *Str*.*mutans* are: bacterial adhesins, acid tolerance, proteases, and putative hemolysins. However, this microorganism is also able to acquire new properties allowing for the expression of virulence in specific environmental conditions [22].

The virulence factors of *C*.*albicans* that might be specifically related to bone damage are not fully recognized, however adhesins and aspartyl proteases or other digestive enzymes might be involved in this process. Also, as it was noticed in a study by Fasetta et al in 2014 that the presence of C. *albicans* augments the production of *Str*.*mutans* EPS in mixed biofilm; moreover coexistence with *C*. *albicans* induces the expression of virulence genes in S. mutans such as *gtfB* and *fabM*, required for attachment to smooth surfaces and survival in low pH, respectively [23]. The last pathogen used in this study, namely *P*.*aeruginosa*, displays an extremely broad spectrum of virulence factors that makes this microbe a very effective opportunistic pathogen. However, all these virulence factors are related with detrimental effects for live eukaryotic cells (including living bone cells) and organic elements of bone. These factors are (among many): serine protease referred to as protease IV; zinc metalloproteases, elastases, flagella and pilli, pyocyanin, or exotoxin A that inhibits host elongation factor 2 (EF2) thereby inhibiting protein synthesis and leading to cell death [24]. Some of these virulence factors are responsible for eukaryotic cell death, while others, such as collagenases, might weaken bone mechanical properties and lead to bone sequestration. The question remains if the same virulence factors are activated when bacteria meet inorganic, hydroxyapatite surfaces. To answer this question, proteomic (secretomic) and genomic studies (based on analysis of mRNA expression) should be performed in the future. Nevertheless, our findings may have important future translational implications for understanding the etiopathogenesis of chronic bone infections. Targeting the pathogenesis of infectious bone disorders will require greater understanding of the interactions between the host innate and acquired immune system, as well as the cellular components of the bone tissue on the one hand, and known biofilm pathogens on the other hand [6]. A disadvantage of presented data is the fact that only reference (and not clinical) microbial strains were used. However, we believe that our proof-of-concept, important from a clinical point of view, will encourage other teams to analyze the observed phenomenon with clinical strains of different pathogenic properties. Although the term ‘osteomyelitis’ suggests by translation an “inflammatory” bone disorder, it remains a condition with an overwhelming complexity of factors and presents a challenge for future research projects to resolve without consideration of all such factors [6]. The application of biofilm theory and methodology in this context will therefore aid in our understanding of osteomyelitis and ultimately inform more targeted antimicrobial therapeutics [25].

## Supporting Information

S1 File Table of pH changes during 7 days of biofilm culture on various media and surfaces. (PDF)

S2 File Number of cells forming biofilm for the various pathogens and substrates tested. (PDF)

## References

[1] Boody BS, Jenkins TJ, Maslak J, Hsu WK, Patel AA. Vertebral Osteomyelitis and Spinal Epidural Abscess: An Evidence-based Review. J Spinal Disord Tech. 2015 7;28(6):E316–27. 10.1097/BSD.0000000000000294 26079841

[2] Sanders J, Mauffrey C. Long bone osteomyelitis in adults: fundamental concepts and current techniques. Orthopedics. 2013 5;36(5):368–75. 10.3928/01477447-20130426-07 23672894

[3] Maffulli N, Papalia R, Zampogna B, Torre G, Albo E, Denaro V. The management of osteomyelitis in the adult. Surgeon. 2016 1 21. pii: S1479-666X(15)00124-9. 10.1016/j.surge.2015.12.005 26805473

[4] Stoodley P, Ehrlich GD, Sedghizadeh PP, Hall-Stoodley L, Baratz ME, Altman DT, et al Orthopaedic biofilm infections. Curr Orthop Pract. 2011 11;22(6):558–563. 10.1097/BCO.0b013e318230efcf 22323927 PMC3272669

[5] Donlan RM, Costerton JW. Biofilms: survival mechanisms of clinically relevant microorganisms. Clin Microbiol Rev. 2002 4;15(2):167–93. 10.1128/CMR.15.2.167-193.2002 11932229 PMC118068

[6] Wolcott RD, Ehrlich GD. Biofilms and chronic infections. J Am Med Assoc. 2008; 299:2682–2684. 10.1001/jama.299.22.2682 18544729

[7] Beck-Broichsitter BE, Smeets R, Heiland M. Current concepts in pathogenesis of acute and chronic osteomyelitis. Curr Opin Infect Dis. 2015 6;28(3):240–5. 10.1097/QCO.0000000000000155 25918958

[8] Khalil H, Williams RJ, Stenbeck G, Henderson B, Meghji S, Nair SP. Invasion of bone cells by Staphylococcus epidermidis. Microbes Infect. 2007 4;9(4):460–5. 10.1016/j.micinf.2007.01.002 17331787

[9] Nair SP, Meghji S, Wilson M, Reddi K, White P, Henderson B. Bacterially induced bone destruction: mechanisms and misconceptions. Infect Immun. 1996 7;64(7):2371–80. 8698454 10.1128/iai.64.7.2371-2380.1996 PMC174085

[10] Junka AF, Szymczyk P, Smutnicka D, Kos M, Smolina I, Bartoszewicz M, et al Microbial biofilms are able to destroy hydroxyapatite in the absence of host immunity in vitro. J Oral Maxillofac Surg. 2015 3;73(3):451–64. 10.1016/j.joms.2014.09.019 25544303 PMC4331275

[11] Li Y, Burne RA. Regulation of the gtfBC and ftf genes of Streptococcus mutans in biofilms in response to pH and carbohydrate. Microbiology. 2001 10;147(Pt 10):2841–8. 10.1099/00221287-147-10-2841 11577162

[12] Assis S, Keenleyside A, Santos AL, Cardoso FA. Bone Diagenesis and its Implication for Disease Diagnosis: The Relevance of Bone Microstructure Analysis for the Study of Past Human Remains. Microsc Microanal. 2015 8;21(4):805–25. 10.1017/S1431927615000768 26169717

[13] Dang H, Lovell CR. Microbial Surface Colonization and Biofilm Development in Marine Environments. Microbiol Mol Biol Rev. 2015 12 23;80(1):91–138. 10.1128/MMBR.00037-15 26700108 PMC4711185

[14] Younes JA, van der Mei HC, van den Heuvel E, Busscher HJ, Reid G. Adhesion forces and coaggregation between vaginal staphylococci and lactobacilli. PLoS One. 2012;7(5):e36917 10.1371/journal.pone.0036917 22629342 PMC3356358

[15] Sedghizadeh PP, Kumar SK, Gorur A, Schaudinn C, Shuler CF, Costerton JW. Microbial biofilms in osteomyelitis of the jaw and osteonecrosis of the jaw secondary to bisphosphonate therapy. J Am Dent Assoc. 2009 10;140(10):1259–65. 19797556 10.14219/jada.archive.2009.0049

[16] Rumpler M, Würger T, Roschger P, Zwettler E, Sturmlechner I, Altmann P, et al Osteoclasts on bone and dentin in vitro: mechanism of trail formation and comparison of resorption behavior. Calcif Tissue Int. 2013 12;93(6):526–39. 10.1007/s00223-013-9786-7 24022329 PMC3827903

[17] Jans M, Nielsen-Marsh C, Smith C, Collins M, Kars H. Characterization of microbial attack on archaeological bone. J Arch Sci. 2004;31:87–95.

[18] Hall-Stoodley L, Stoodley P. Evolving concepts in biofilm infections. Cell Microbiol. 2009; 11:1034–1043. 10.1111/j.1462-5822.2009.01323.x 19374653

[19] Burne RA, Marquis RE. Biofilm acid/base physiology and gene expression in oral bacteria. Methods Enzymol. 2001;337:403–15. 11398446 10.1016/s0076-6879(01)37029-5

[20] Paterson JS, Ogden S, Smith RJ, Delpin MW, Mitchell JG, Quinton JS. Surface modification of an organic hessian substrate leads to shifts in bacterial biofilm community composition and abundance. J Biotechnol. 2016 2 10;219:90–7. 10.1016/j.jbiotec.2015.12.033 26721183

[21] Cassat JE, Hammer ND, Campbell JP, Benson MA, Perrien DS, Mrak LN, et al A secreted bacterial protease tailors the Staphylococcus aureus virulence repertoire to modulate bone remodeling during osteomyelitis. Cell Host Microbe. 2013 6 12;13(6):759–72. 10.1016/j.chom.2013.05.003 23768499 PMC3721972

[22] Krzyściak W, Jurczak A, Kościelniak D, Bystrowska B, Skalniak A. The virulence of Streptococcus mutans and the ability to form biofilms. Eur J Clin Microbiol Infect Dis. 2014 4;33(4):499–515. 10.1007/s10096-013-1993-7 24154653 PMC3953549

[23] Falsetta ML, Klein MI, Colonne PM, Scott-Anne K, Gregoire S, Pai CH, et al Symbiotic Relationship between Streptococcus mutans and Candida albicans Synergizes Virulence of Plaque Biofilms In Vivo. Infect Immun. 2014 5; 82(5): 1968–1981. 10.1128/IAI.00087-14 24566629 PMC3993459

[24] Gellatly SL, Hancock RE. Pseudomonas aeruginosa: new insights into pathogenesis and host defenses. Pathog Dis. 2013 4;67(3):159–73. 10.1111/2049-632X.12033 23620179

[25] Brady RA, Leid JG, Calhoun JH, Costerton JW, Shirtliff ME. Osteomyelitis and the role of biofilms in chronic infection. FEMS Immunol Med Microbiol. 2008 1;52(1):13–22. 10.1111/j.1574-695X.2007.00357.x 18081847

