# Supplementary material for: Bad to the Bone: On In Vitro and Ex Vivo Microbial Biofilm Ability to Directly Destroy Colonized Bone Surfaces without Participation of Host Immunity or Osteoclastogenesis
Source: PLoS One. 2017 Jan 11;12(1):e0169565. doi: 10.1371/journal.pone.0169565 (PMC5226730; doi:10.1371/journal.pone.0169565)
Supplement: S1 File — (PDF) [file pone.0169565.s001.pdf]

			pH values in 7 days of culture						
**Microorganism(s)**	**medium:**	**surface:**	**1st day**	**2nd day**	**3rd day**	**4th day**	**5th day**	**6th day**	**7th day**
S.aureus ATTC 6538	TSB	polystyrene	pH=6	7	8	9	10	10	9
		HA disc	6	6	7	7	8	8	8
		Rat jaw	6.5	6.5	7	8	8	8	8
	TSB + sucrose	polystyrene	6	7	6	7	6	6	6
		HA disc	6	5	5	6	7	6	5
		Rat Jaw	6.5	6	6	6	6	6	6
	saliva	polystyrene	6	7	7	8	9	10	10
		HA disc	6	6	6	6	6	6	6
		Rat jaw	8	8	8	9	9	9	9
	saliva+ sucrose	polystyrene	6	6	6	7	6	6	6
		HA disc	6	6	6	6	6	6	6
		Rat jaw	7	8	8	9	9	9	9
	**medium:**	**surface:**							
Streptococcus mutans ATTC 25175	BHI	polystyrene	5	4	5	5	5	5	5
		HA disc	5	4	5	5	4	4	5
		Rat jaw	6	6	6	6	6	6	6
	BHI + sucrose	Polystyrene	5	4	4	5	5	5	5
		HA disc	5	4	4	4	5	5	5
		Rat jaw	5	5	5	5	5	5	5
	saliva	polystyrene	4	5	4	5	5	5	5
		HA disc	5	5	4	5	5	4	5
		Rat jaw	6	6	6	6	6	6	6
	Saliva + sucrose	polystyrene	3	4	5	5	5	5	5
		HA disc	5	5	4	5	4	4	5
		Rat Jaw	6	5	6	5	6	5	5

			pH values in 7 days of culture						
Candida albicans ATTC 10231	**medium**	**surface**	**1st day**	**2nd day**	**3rd day**	**4th day**	**5th day**	**6th day**	**7th day**
	TSB	polystyrene	6	7	7	8	8	8	9
		HA disc	7	8	8	6	6	6	5
		Rat jaw	7	7	8	8	8	8	8
	TSB + sucrose	polystyrene	6	7	6	6	6	8	6
		HA disc	7	6	6	6	6	6	5
		Rat jaw	7.5	8	8	9	9	9	9
	saliva	polystyrene	6	7	7	8	8	8	9
		HA disc	9	9	9	10	9	10	10
		Rat jaw	7.5	8	8	9	9	9	9
	Saliva + sucrose	polystyrene	6	7	8	8	7	7	8
		HA disc	9	7	10	10	9	10	10
		Rat Jaw	7	8	9	9	9	9	9
	**Medium**	**Surface**							
P.aeruginosa ATTC 15442	**TSB**	polystyrene	8	8	8	7	8	9	9
		HA disc	8	9	9	9	8	9	9
		Rat jaw	7	8	8	9	9	8	8
	**TSB + sucrose**	polystyrene	8	8	8	8	8	9	9
		HA disc	8	8	8	7	8	9	9
		Rat Jaw	7.5	8	8	9	9	9	9
	**saliva**	polystyrene	8	8	8	8	8	9	9
		HA disc	8	8	8	8	9	9	10
		Rat jaw							
	**Saliva + sucrose**	polystyrene	8	8	8	8	8	9	9
		HA disc	8	7	8	8	9	9	10
		Rat jaw	8	8	9	9	9	9	9
