# Supplementary material for: Bad to the Bone: On In Vitro and Ex Vivo Microbial Biofilm Ability to Directly Destroy Colonized Bone Surfaces without Participation of Host Immunity or Osteoclastogenesis
Source: PLoS One. 2017 Jan 11;12(1):e0169565. doi: 10.1371/journal.pone.0169565 (PMC5226730; doi:10.1371/journal.pone.0169565)
Supplement: S2 File — (PDF) [file pone.0169565.s002.pdf]

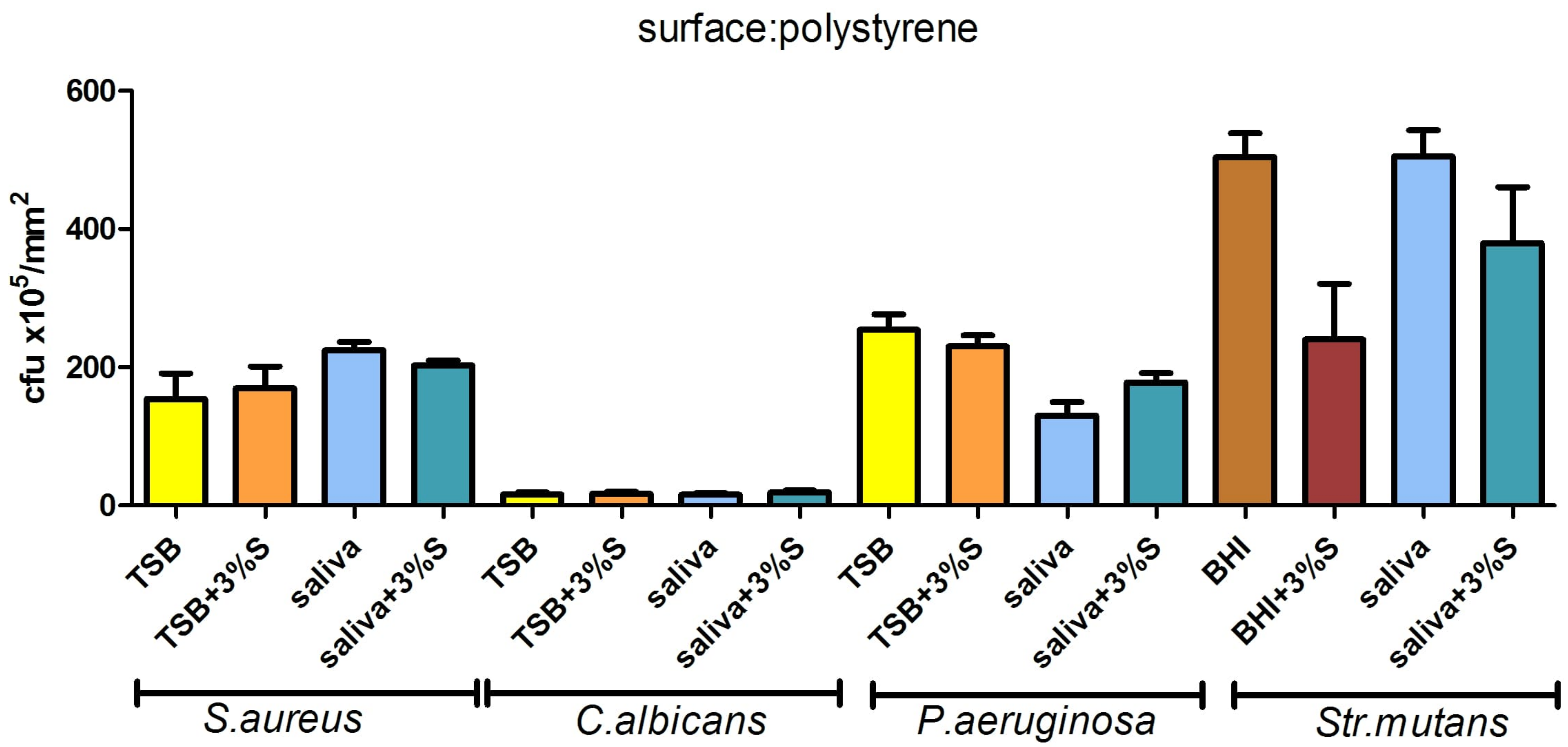
surface:polystyrene
cfu x10^5/mm^2
600
400
200
0
TSB
TSB+3%S
saliva
saliva+3%S
TSB
TSB+3%S
saliva
saliva+3%S
TSB
TSB+3%S
saliva
saliva+3%S
BHI
BHI+3%S
saliva
saliva+3%S
S.aureus
C.albicans
P.aeruginosa
Str.mutans

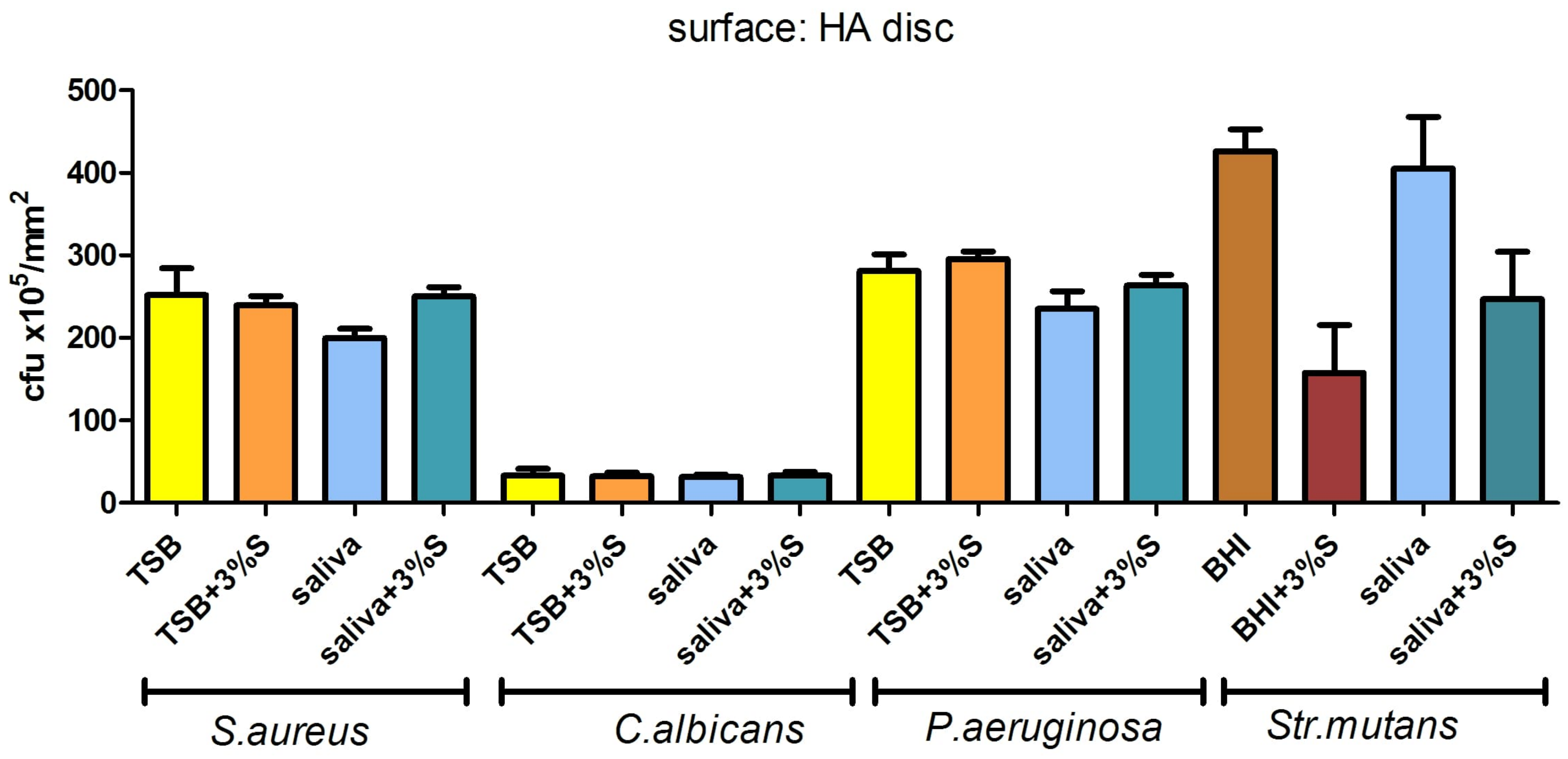

surface: HA disc
cfu x10^5/mm^2
0
100
200
300
400
500
TSB
TSB+3%S
saliva
saliva+3%S
TSB
TSB+3%S
saliva
saliva+3%S
TSB
TSB+3%S
saliva
saliva+3%S
BHI
BHI+3%S
saliva
saliva+3%S
S.aureus
C.albicans
P.aeruginosa
Str.mutans

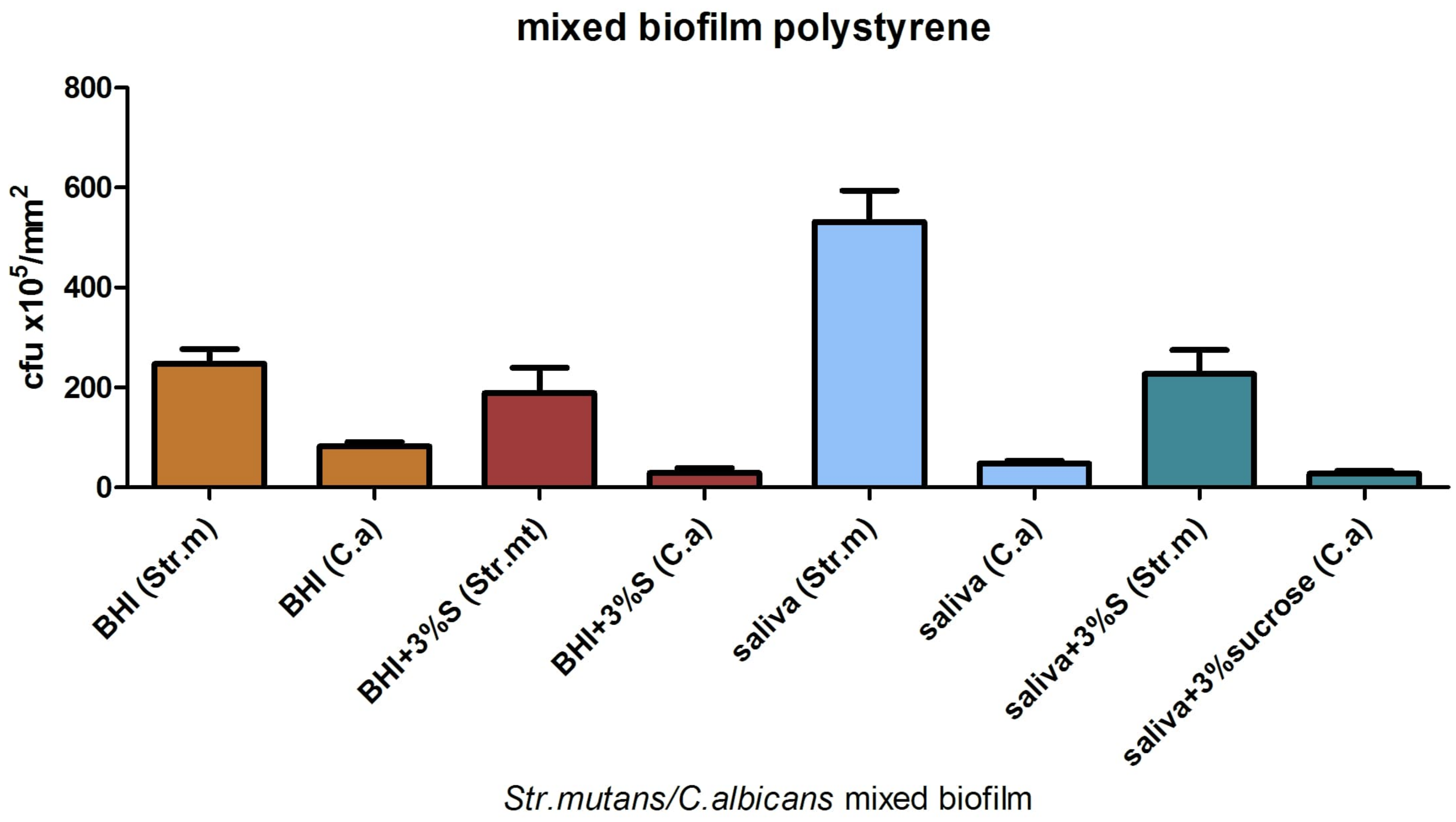
mixed biofilm polystyrene
cfu x10^5/mm^2
800
600
400
200
0
BHI (Str.m)
BHI (C.a)
BHI+3%S (Str.mt)
BHI+3%S (C.a)
saliva (Str.m)
saliva (C.a)
saliva+3%S (Str.m)
saliva+3%sucrose (C.a)
Str.mutans/C.albicans mixed biofilm

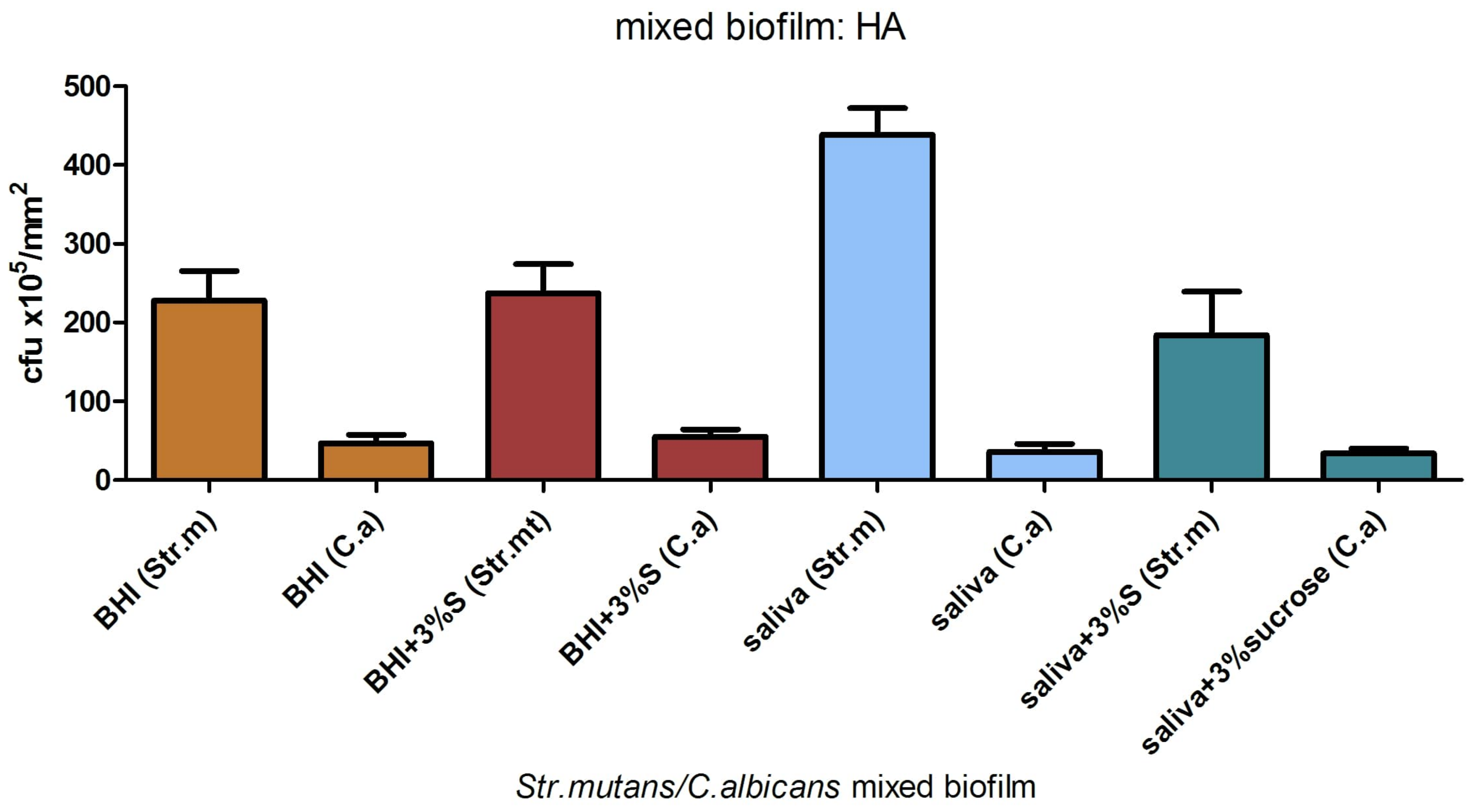

mixed biofilm: HA
cfu x10^5/mm^2
500
400
300
200
100
0
BHI (Str.m)
BHI (C.a)
BHI+3%S (Str.mt)
BHI+3%S (C.a)
saliva (Str.m)
saliva (C.a)
saliva+3%S (Str.m)
saliva+3%sucrose (C.a)
Str.mutans/C.albicans mixed biofilm

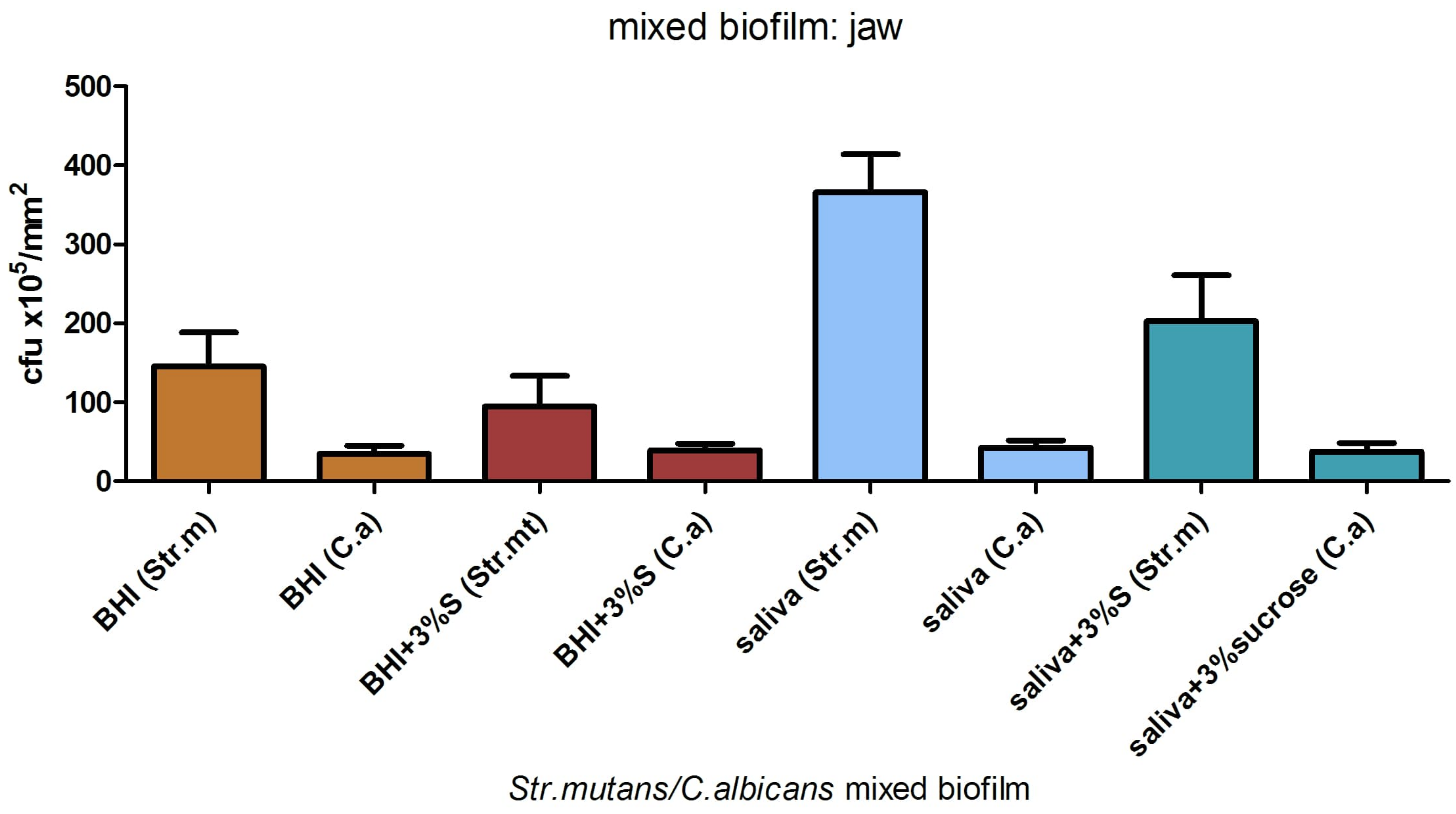
mixed biofilm: jaw
cfu x10^5/mm^2
500
400
300
200
100
0
BHI (Str.m)
BHI (C.a)
BHI+3%S (Str.mt)
BHI+3%S (C.a)
saliva (Str.m)
saliva (C.a)
saliva+3%S (Str.m)
saliva+3%sucrose (C.a)
Str.mutans/C.albicans mixed biofilm
